# Monomer conversion, dimensional stability, strength, modulus, surface apatite precipitation and wear of novel, reactive calcium phosphate and polylysine-containing dental composites

**DOI:** 10.1371/journal.pone.0187757

**Published:** 2017-11-14

**Authors:** Kanokrat Kangwankai, Sarah Sani, Piyaphong Panpisut, Wendy Xia, Paul Ashley, Haralampos Petridis, Anne Margaret Young

**Affiliations:** 1 Department of Restorative Dentistry, Unit of Prosthodontics, UCL Eastman Dental Institute, London, United Kingdom; 2 Unit of Orthodontics, UCL Eastman Dental Institute, London, United Kingdom; 3 Department of Biomaterials and Tissue Engineering, UCL Eastman Dental Institute, London, United Kingdom; 4 Faculty of Dentistry, Thammasat University, Pathumthani, Thailand; 5 Unit of Paediatric Dentistry, UCL Eastman Dental Institute, London, United Kingdom; Institute of Materials Science, GERMANY

## Abstract

**Purpose:**

The aim was to assess monomer conversion, dimensional stability, flexural strength / modulus, surface apatite precipitation and wear of mono / tri calcium phosphate (CaP) and polylysine (PLS)—containing dental composites. These were formulated using a new, high molecular weight, fluid monomer phase that requires no polymerisation activator.

**Materials and methods:**

Urethane and Polypropylene Glycol Dimethacrylates were combined with low levels of an adhesion promoting monomer and a light activated initiator. This liquid was mixed with a hybrid glass containing either 10 wt% CaP and 1 wt% PLS (F1) or 20 wt% CaP and 2 wt% PLS (F2). Powder to liquid mass ratio was 5:1. Commercial controls included Gradia Direct Posterior (GD) and Filtek Z250 (FZ). Monomer conversion and polymerisation shrinkage were calculated using Fourier Transform Infrared (FTIR). Subsequent volume increases in water over 7 weeks were determined using gravimetric studies. Biaxial flexural strength (BFS) / modulus (BFM) reduction and surface apatite precipitation upon 1 and 4 weeks immersion in water versus simulated body fluid (SBF) were assessed using a mechanical testing frame and scanning electron microscope (SEM). Mass / volume loss and surface roughness (R_a_) following 7 weeks water immersion and subsequent accelerated tooth-brush abrasion were examined using gravimetric studies and profilometer.

**Results:**

F1 and F2 exhibited much higher monomer conversion (72%) than FZ (54%) and low calculated polymerization shrinkage (2.2 vol%). Final hygroscopic expansions decreased in the order; F2 (3.5 vol%) > F1 (1.8 vol%) ~ Z250 (1.6 vol%) > Gradia (1.0 vol%). BFS and BFM were unaffected by storage medium type. Average BFS / BFM upon 4 weeks immersion reduced from 144 MPa / 8 GPa to 107 MPa / 5 GPa for F1 and 105 MPa / 6 GPa to 82 MPa / 4 GPa for F2. Much of this change was observed in the first week of immersion when water sorption rate was high. Surface apatite layers were incomplete at 1 week, but around 2 and 15 micron thick for F1 and F2 respectively following 4 weeks in SBF. Mass and volume loss following wear were equal. Average results for F1 (0.5%), F2 (0.7%), and FZ (0.5%) were comparable but lower than that of GD (1%). R_a_, however, decreased in the order; F1 (15 μm) > F2 (11 μm) > GD (9 μm) > FZ (5 μm).

**Conclusions:**

High monomer conversion in combination with large monomer size and lack of amine activator should improve cytocompatibility of the new composites. High monomer molecular weight and powder content enables low polymerisation shrinkage despite high conversion. Increasing active filler provides enhanced swelling to balance shrinkage, which, in combination with greater surface apatite precipitation, may help seal gaps and reduce bacterial microleakage. High monomer conversion also ensures competitive mechanical / wear characteristics despite enhanced water sorption. Furthermore, increased active filler could help reduce surface roughness upon wear.

## Introduction

With the advent of the Minamata Convention [1], amalgam as a dental restorative material is being phased out. The alternative composite materials, however, generally have higher annual failure rates [2]. Composite restoration failure is primarily due to polymerization shrinkage which can lead to microgap formation that allows nutrient and / or bacterial penetration [3]. Bacteria can subseqently enable continuation of tooth apatite dissolution (demineralization) and decay beneath the composite restoration (recurrent or secondary caries) [4].

Composites containing antibacterial chlorhexidine diacetate (CHX) and reactive calcium phosphate (CaP) fillers have been developed to reduce bacterial microleakage [5–7]. The CaP consists of mono-calcium phosphate monohydrate (MCPM) and beta-tricalcium phosphate (β-TCP). These react together with absorbed water to form higher volume dicalcium phosphate precipitates that may enable material expansion to compensate polymerization shrinkage. In contact with simulated body fluids, these precipitates can transform into low density apatite potentially sealing cracks in the material or at the restoration / dentin interface, and remineralize the demineralized dentin [8].

A concern with the above described composites is the use of the low molecular weight, high shrinkage, triethylene glycol dimethacrylate (TEGDMA) diluent monomer [9]. Additionally, the above composites contain the tertiary amine activator, dimethyl-*p*-toluidine (DMPT). This amine functions as an electron donor or reducing agent which can increase production of free radicals thus increasing final monomer conversion of dental composites [10]. Low concentrations of this amine, however, exhibit both cyto- and geno-toxicity effects [11, 12]. A recent study has shown that replacing TEGDMA by higher molecular weight polypropylene glycol dimethacrylate (PPGDMA) can improve monomer conversion in conventional dental composites which may reduce the need for any amine activator[13]. PPGDMA high molecular weight also ensures low shrinkage and improved monomer cytocompatibility.

A further problem with the above CaP composites has been the use of CHX. CHX is a cationic bisbiguanide with excellent antimicrobial action [14]. It has been demonstrated, however, that CHX concentrations as low as 0.06% can be toxic to cultured odontoblast-like cells [15]. Additionally, there have been recent reports of anaphylactic shock and death of patients following use of CHX mouthwash [16]. Furthermore, to enable high CHX release from composites, hydrophilic components need to be added to promote water sorption [5, 7]. The FDA approved food preservative ε-poly-L-lysine (PLS) is a possible alternative antimicrobial agent. This naturally synthesized compound exhibits low toxicity to human cells and has a wide antimicrobial spectrum [17]. Furthermore, PLS can readily be released from hydrophobic composites and enhances apatite precipitation on CaP composites [18].

Whilst water sorption induced expansion may beneficially balance polymerisation shrinkage and improve release of active ingredients such as polylysine or calcium and phosphate ions, it can also reduce composite strength and modulus [5, 18, 19]. Additionally, agent release may lead to surface pores, reduce wear resistance, and enhance surface roughness [20] which facilitates biofilm accumulation [21].

This study therefore determines monomer conversion, dimensional stability, surface apatite precipitation and mechanical properties of CaP, PLS and PPGDMA—containing dental composites with no amine activator. Furthermore, mass / volume loss and surface roughness (R_a_) after accelerated wear testing are investigated.

## Materials and methods

### Composite paste and disc preparation

The experimental composites were prepared using a powder to liquid mass ratio of 5: 1. The monomer phase of all experimental composites contained 72 wt% urethane dimethacrylate (UDMA) (DMG, Hamburg, Germany) and 24 wt% PPGDMA (Polysciences Inc, Hirschberg an der Bergstrasse, Germany). 3 wt% 4-methacryloxyethyl trimellitic anhydride (4-META) (Polysciences Inc, Hirschberg an der Bergstrasse, Germany) was added. This may help bind the monomer phase to amine groups and calcium ions in both the composite and dentin. 1 wt% camphorquinone (CQ) (Polysciences Inc, Hirschberg an der Bergstrasse, Germany) was included as initiator.

**Fig 1:**
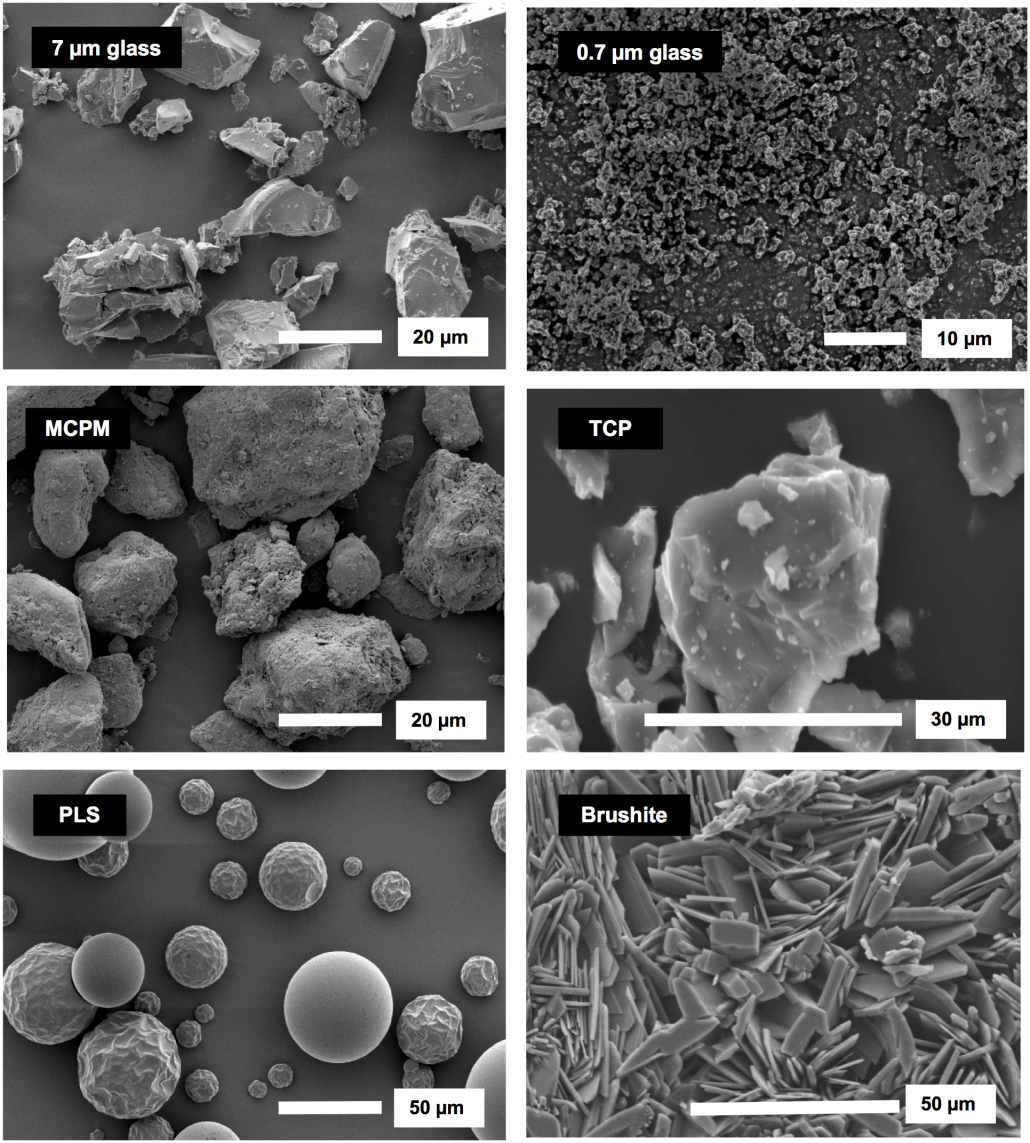
SEM images of filler particles and brushite.

The glass fillers consisted of 40 nm silica (Azelis, Hertford, UK) combined with two silane treated aluminosilicate glasses (DMG, Hamburg, Germany) with average diameters of 0.7 μm and 7 μm. The mass ratio of 40 nm: 0.7 μm: 7 μm fillers was 1:3:6 to maximize packing. MCPM (53 μm, batch MCP-B26, HiMed, Old Bethpage, NY, USA), β-TCP (34 μm, batch P292 S, Plasma Biotal, Buxton, UK) and PLS (4700 g/mol, 20–50 μm, batch 09010203, Handary S.A., Brussels, Belgium) were used as received. SEM images of filler particles are provided in Fig 1 in addition to an image of brushite crystals formed by mixing MCPM and β-TCP with water.

Formulation 1 (F1) and 2 (F2) powders contained 89 wt% and 78 wt% glass respectively. F1 powder also contained 5 wt% MCPM, 5 wt% β-TCP, and 1 wt% PLS whilst F2 powder had 10 wt% MCPM, 10 wt% β-TCP, and 2 wt% PLS. The powder and liquid phase were mixed using a planetary mixer (SpeedMixer, DAC 150.1 FVZ, Hauschild Engineering, Germany) at 3500 rpm for 10 s followed by 2000 rpm for 2 min. Qualitative evaluation of consistency and colour of the mixed pastes were examined visually. Two commercially available dental composites were used as controls; Filtek Z250 (Lot number N519660, Shade B3, 3M ESPE, St Paul, MN, USA) and Gradia Direct Posterior (Lot number 1308132, Shade P-A2, GC, Tokyo, Japan).

Disc specimens were prepared using metal circlips (15 or 10 mm internal diameter and 1 mm in thickness) as moulds. The composite pastes were placed in a circlip and covered with acetate sheets on top and bottom sides. They were then light cured by an LED light curing unit (1,100–1,330 mW/cm^2^, Demi Plus, Kerr, USA) for 40 s with a circular motion on both sides. Specimens were left at room temperature for at least 24 hr to ensure completion of polymerization. After removal from the circlip, any excesses were trimmed. Specimens were then stored in tubes containing 10 cm^3^ of deionized water or simulated body fluid (SBF) prepared according to BS ISO 23317:2012 [22] at 37°C until the required test time.

### Monomer conversion and calculated polymerization shrinkage

Monomer conversion of composite pastes (n = 5) at 25°C was assessed using an FTIR (Perkin-Elmer Series 2000, Beaconsfield, UK) equipped with a golden gate Attenuated Total Reflectance (ATR) accessory (3000 Series RS232,Specac Ltd., Kent, UK) [18]. Briefly, the uncured composite paste was placed in a circlip (1 mm thick and 10 mm in diameter) centred over the ATR diamond. The paste was covered with an acetate sheet and cured by an LED light curing unit as above from the top surface for 40 s. FTIR spectra in the region of 400–1700 cm^-1^ with a resolution of 8 cm^-1^ were recorded from the bottom of the sample for 20 min. The monomer conversion versus time was calculated using the following equation [18].

$$C(\%)\;=\;\frac{100({\Delta A}_{0}-{\Delta A}_{t})}{{\Delta A}_{0}}$$ (1)

Where Δ*A*_0_ and Δ*A*_*t*_ were the absorbance of the C-O peak (1320 cm^-1^) above background level at 1335 cm^-1^ initially and after time *t*. Final conversion was obtained from the y intercept of conversion versus inverse time.

Final polymerization shrinkage percentage (*φ*) was additionally calculated using the following equation [18].

$$\varphi (\%)=e(M_{f})C\rho \;\sum_{i}\frac{n_{i}x_{i}}{W_{i}}$$ (2)

Where; *e*, volumetric shrinkage (23 cm^3^/mol) generated by one mole of polymerizing methacrylate (C = C) group; *M*_*f*_, monomer fraction; *C*, final monomer conversion percentage; *ρ*, composite density; *n*_*i*_, he number of C = C bonds per molecule (2); *W*_*i*_, molecular weight (g/mol) of each monomer; *x*_*i*_, mass fraction of each monomer.

### Mass and volume changes

Mass and volume change (n = 3), after 1, 2, 4, 24, 48 hr and 1, 2, 3, 4, 5, 6, 7 weeks in deionized water of 15 mm diameter discs were determined using a four-figure balance with an attached density determination kit (Mettler-Toledo AG, Greifensee, Switzerland) [5]. The percentage mass (ΔM) and volume increases (ΔV) at each time point were calculated using;

$$\Delta M\;(\%)\;=\;\frac{100[\;M_{t}-M_{0}]}{M_{0}}$$ (3)

$$\Delta V(\%)\;=\;\frac{100[\;V_{t}-V_{0}]}{V_{0}}$$ (4)

Where *M*_*t*_ and *V*_*t*_ were the mass and volume at time *t* after immersion and *M*_0_ and *V*_0_ were the initial mass and volume [7].

### Biaxial flexural strength (BFS) and modulus (BFM)

Biaxial flexural strength (BFS) and modulus (BFM) (n = 8) of 10 mm diameter discs were determined after 0, 1 and 4 weeks, water or SBF storage, using a ‘Ball on ring’ jig and mechanical testing frame (Shimadzu AGSX, Tokyo, Japan) at a cross-head speed of 1 mm/min. The maximum force (*P)* was recorded and BFS (*σ*) and BFM (*E*) were calculated using Eqs 5 and 6, respectively [18].

$$\sigma \;=\;\frac{P}{t^{2}}[(1+v)(0.485\;ln\;(\frac{a}{t})+0.52)+0.48]$$ (5)

Where *a* is support radius (4 mm), *t* is specimen thickness, and *v* is Poisson’s ratio (0.3).

$$E\;=\;(\frac{\Delta P}{\Delta d})\;\times \;(\frac{\beta_{c}a^{2}}{t^{3}})$$ (6)

Where $\frac{\Delta P}{\Delta d}$ is gradient of force versus displacement curve and *β*_*c*_ is center deflection function (0.5024) [13].

### Surface apatite formation

Apatite precipitation (n = 1) on 10 mm diameter discs after 1 or 4 weeks immersion in SBF was assessed. After blot drying, specimens were coated with gold-palladium alloy and examined using scanning electron microscopy (SEM, Phillip XL-30, Eindhoven, The Netherlands) with a primary beam energy of 5 kV and a current of approximately 200 pA. Additionally, elemental analysis was undertaken using EDX (Energy Dispersive X-ray Analysis, Inca X-sight 6650 detector, Oxford Instrument, UK) with 20 kV accelerating voltage.

### Accelerated wear testing

**Fig 2:**
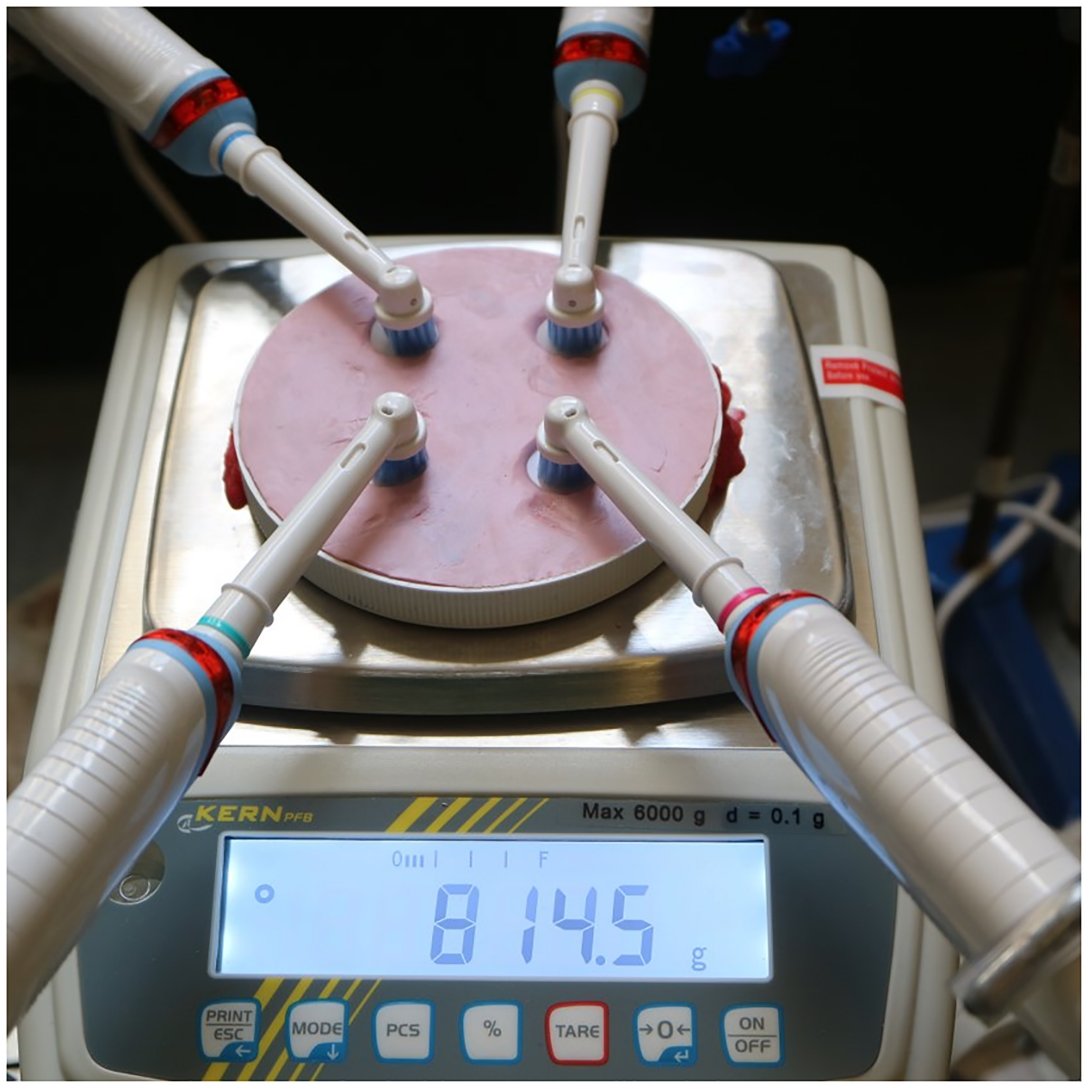
Tooth brushing apparatus.

For the wear test (n = 8), 15 mm disc samples were pre-aged in deionized water for 7 weeks. Discs were subsequently placed in a custom-made silicone mould attached with a digital scale that could hold 4 specimens (Fig 2). Flat-bristle toothbrush heads (Oral-B Sensitive, Braun GmbH, Kronberg, Germany) connected to rotating electric toothbrushes (Oral-B Professional care 1000, Braun GmbH, Kronberg, Germany) were placed on the specimen’s surface [23]. The mould was lifted until a force of 8 N (816 g) was achieved so that each sample was loaded with 2 N [24]. A total of 20 min brushing with pumice (diameter of ~ 100 μm, Skillbond1, Skillbond Direct, High Wycombe, UK) was undertaken. Specimens were subsequently rinsed and cleaned in an ultrasonic bath for 1 min to remove debris.

Percentage mass and volume loss from wear (n = 8) were determined from mass and volume before and after brushing using Eqs 3 and 4. A two-dimensional arithmetic average surface roughness (R_a_) [25] of composite surfaces before (R_a_ initial) and after (R_a_ final) the brushing test was also determined using a Laser Profilometer (ProScan 1000, Scantron, Sommerset, UK) (n = 8). For each sample, the profilometer laser scanned 600 lines, 0.005 mm apart in 2 areas of 3 mm x 3 mm in the most abraded regions. R_a_ was then determined using 5 lines of 3 mm length, each 0.5 mm apart in both x and y directions within each area. With 10 lines per area, this gave a total of 20 readings for each specimen. The surface and bulk topography and chemistry (n = 1) before and after water sorption, wear, and specimen fracture were compared using SEM with EDX as in 2.5.

### Statistical analysis

Descriptive statistics include mean values as point estimates and standard deviation of measured values. Raw data are provided in S1 File. Data were analyzed using SPSS^®^ Version 24 for Windows (IBM, NY, USA). Data were subjected to Levene's test of homogeneity of variance. Volume loss, initial R_a_, and final R_a_ (with variances equal) were compared using one-way analysis of variance (ANOVA) and Tukey’s post-hoc multiple comparison. Monomer conversion, BFS and BFM, and mass loss (variances unequal) were analyzed using Kruskal-Wallis test followed by Dunnett T3 post-hoc multiple comparison [26]. The significance value was set at *p* = 0.05.

## Results

### Paste appearance

The consistencies of the mixed experimental composites were comparable with those of the commercial composites. After curing, their colour changed from pale yellow to white.

### Monomer conversion and calculated polymerization shrinkage

Monomer conversion of F1 (72 ± 1%) and F2 (72 ± 4%) were comparable (*p* > 0.05) and very much higher than that of Z250 (54 ± 3%). The presence of pre-polymerized fillers in Gradia made FTIR unsuitable for calculating monomer conversion. F1 and F2 calculated shrinkages were 2.24 ± 0.10 vol% and 2.18 ± 0.12 vol% respectively. The polymerization shrinkage of Z250 was not calculated as the exact composition was unknown.

### Mass and volume change in water

**Fig 3:**
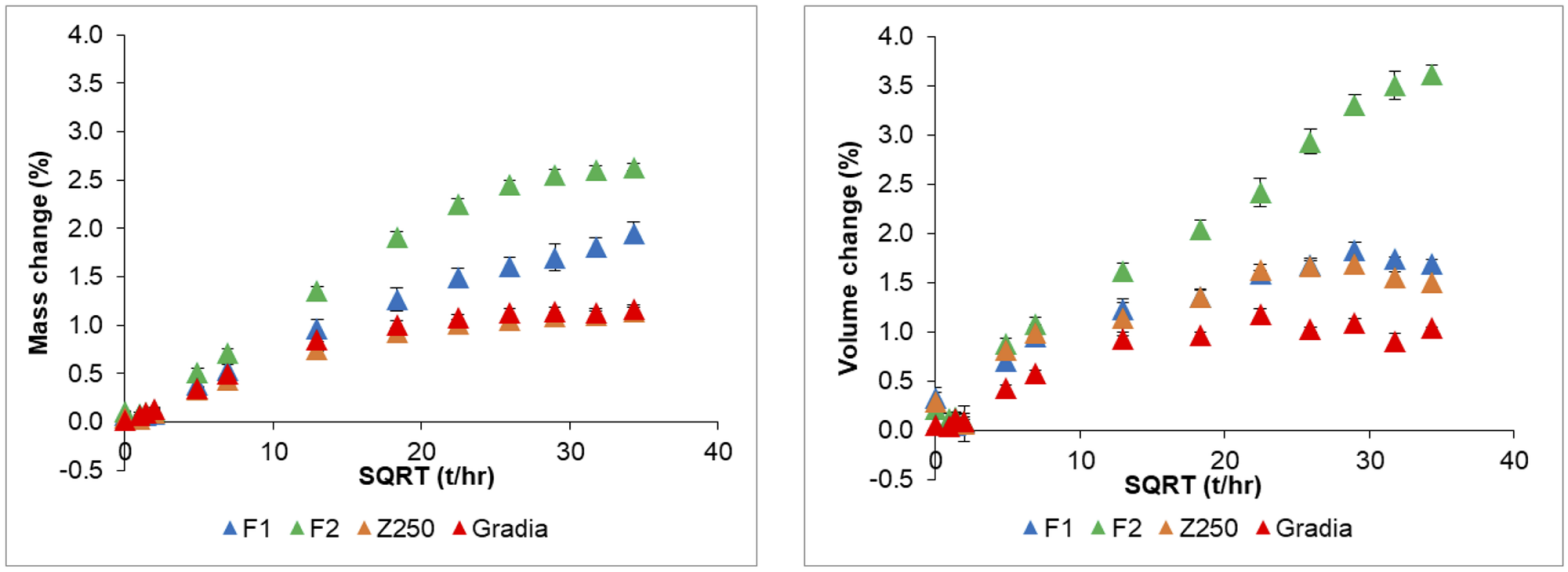
Composite disc A) mass and B) volume changes in deionized water as a function of square root (SQRT) of time, t, over 7 weeks. Error bars are SD (n = 3).

Early percentage mass and volume change increased linearly with square root of time for all formulations (Fig 3). Mass changes began to plateau at 1 week for Z250 and Gradia and at 3 weeks for F1 and F2. Volume changes levelled off slightly earlier for all formulations except F2. Averaged late time (5–7 weeks) mass increases were for F2 (2.60 ± 0.04 wt%) > F1 (1.82 ± 0.13 wt%) > Z250 (1.11 ± 0.02 wt%) ~ Gradia (1.14 ± 0.05 wt%). Averaged volume changes in this period were for F2 (3.48 ± 0.15 vol%) > F1 (1.76 ± 0.07 vol%) ~ Z250 (1.59 ± 0.10 vol%) > Gradia (1.02 ± 0.09 vol%).

### Biaxial flexural strength (BFS) and modulus (BFM)

**Fig 4:**
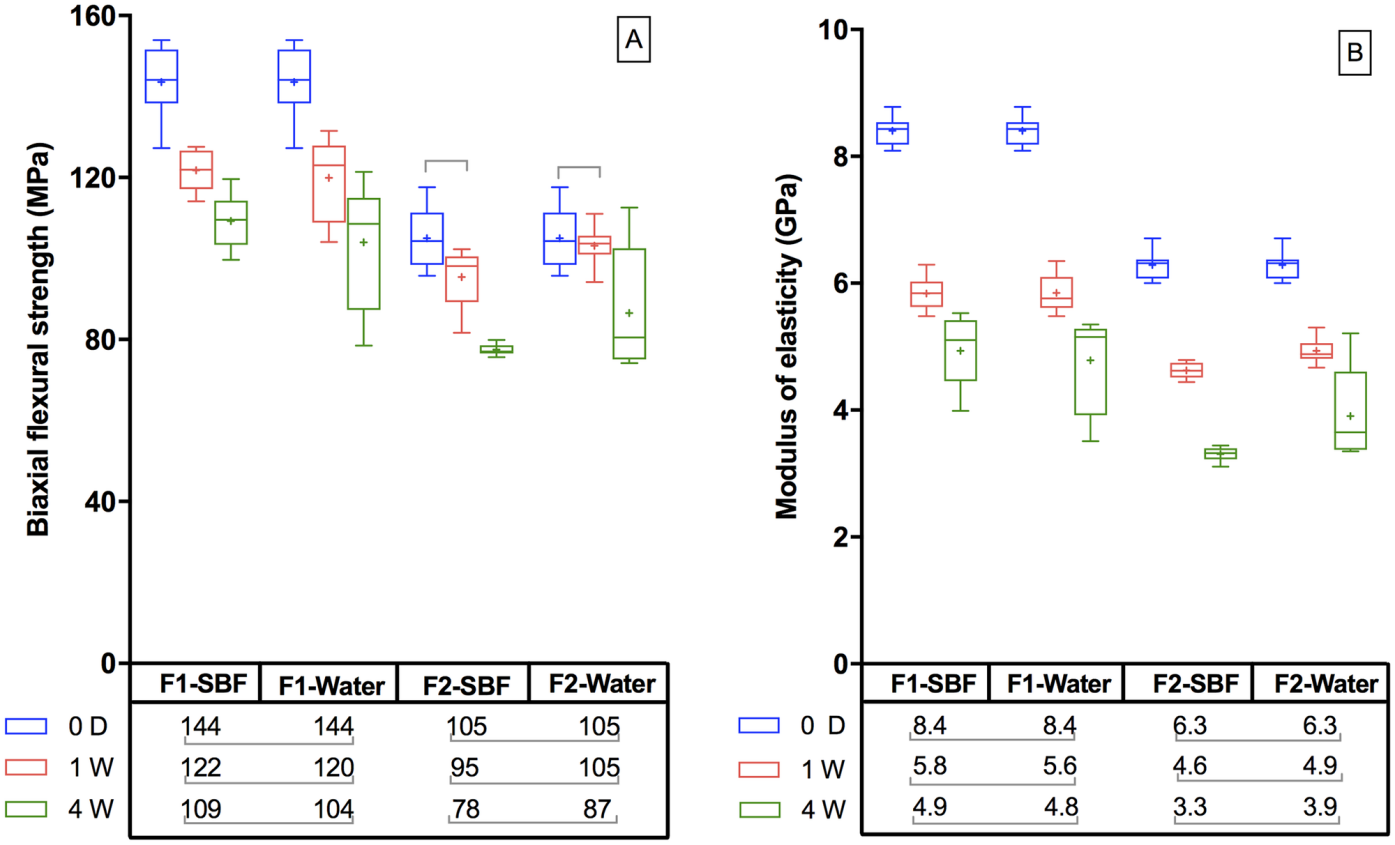
A) BFS and B) BFM of experimental composites before and after immersion in deionized water or SBF for up to 4 weeks. The boxes represent the first quartile (Q1) to the third quartile (Q3), the horizontal lines in the box represent the median, the whiskers represent the maximum and minimum values, and “+” represents the mean value (n = 8). Lines indicate no significant differences (*p*>0.05).

Changing the storage medium from deionized water to SBF had on average negligible effect on BFS and BFM (Fig 4). Levels of decline in BFS and BFM between 1 and 4 weeks were comparable to those seen in the first week. Generally, BFS and BFM were higher and their decline with time levelled more quickly with F1 than with F2. Upon 4 weeks immersion, average BFS was reduced from 143 to 106 MPa for F1 and from 105 to 82 MPa for F2. Average BFM was reduced from 8.4 to 4.9 GPa for F1 and 6.3 to 3.6 GPa for F2.

### Apatite formation in SBF

**Fig 5:**
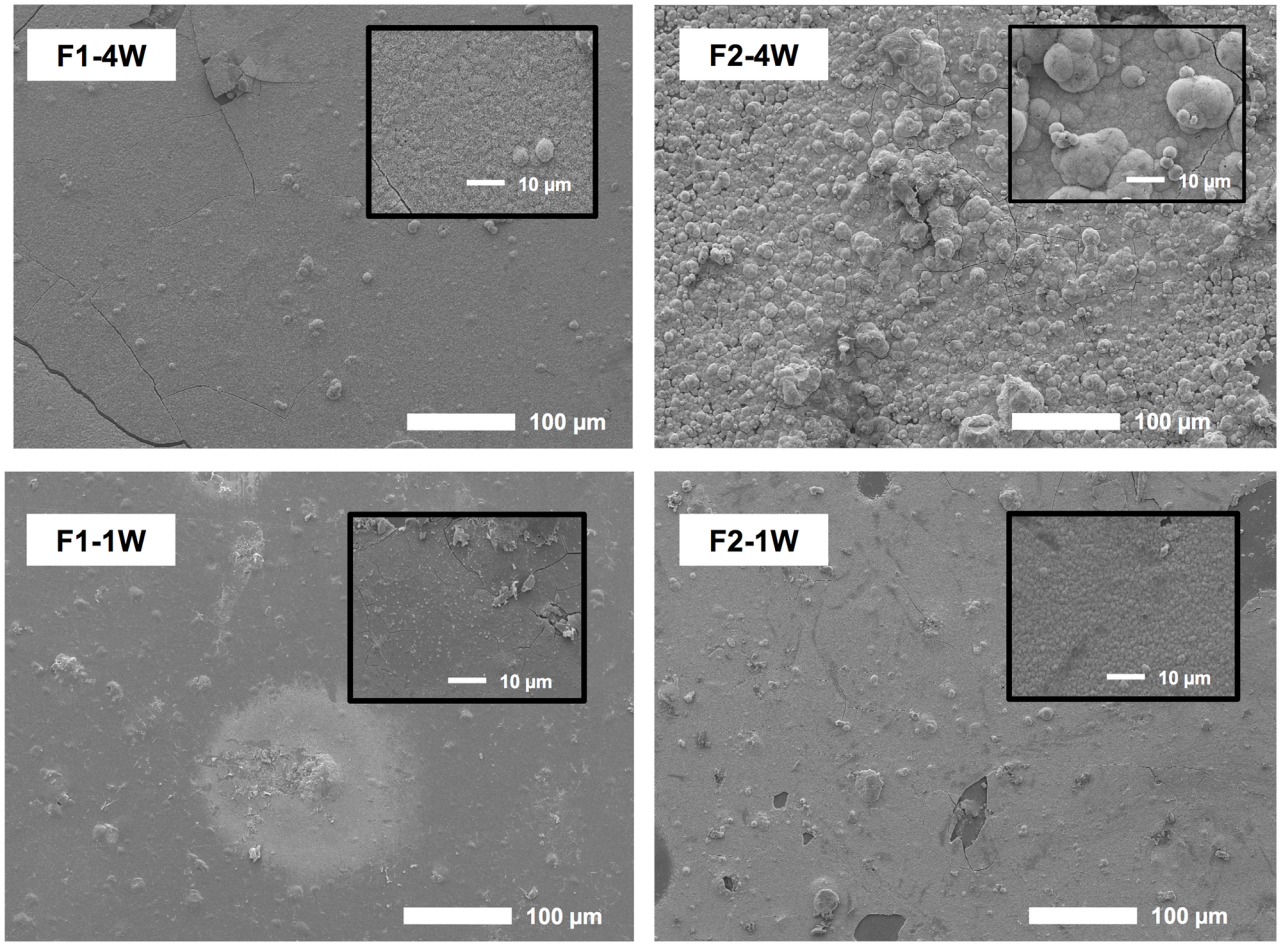
SEM images of HA formation on composite surface after 1 and 4 weeks immersion in SBF.

After 1 week immersion in SBF, an apatite like globular precipitate was detected on F1 (Fig 5). With F1, the layer was patchy at 1 week but more homogeneous by 4 weeks. From the size of the precipitate globules, the layer thickness was estimated to be ~2 μm at this later time. With F2, layers of ~ 2 and 15 μm were observed at 1 and 4 weeks respectively. Surface Ca/P ratio increased from 1 originally to 1.3 in thicker apatite layers. No precipitation was detected on the commercial materials or on specimens stored in water.

### Accelerated wear testing

**Fig 6:**
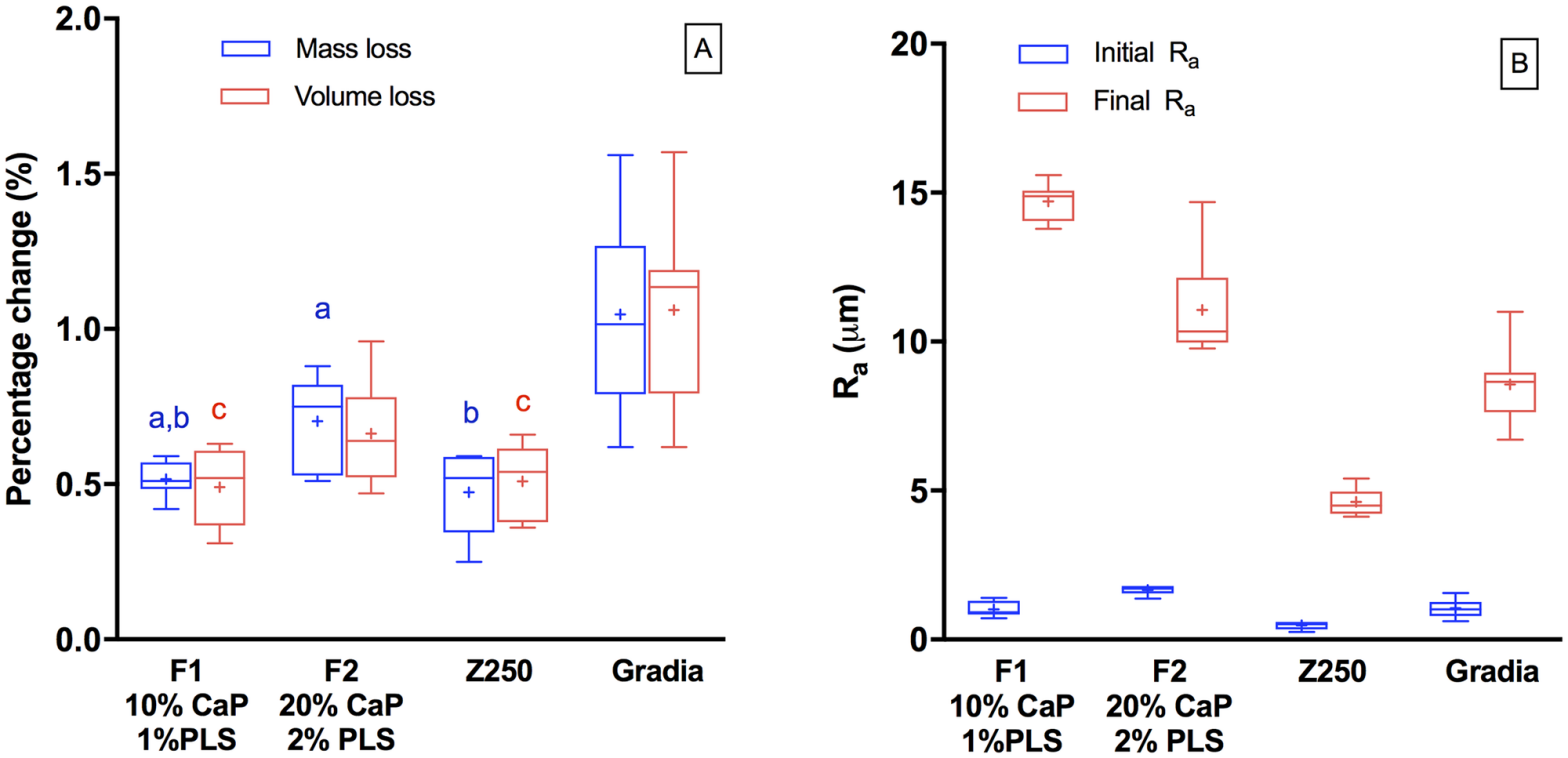
A) Percentage mass and volume loss after tooth brushing for 20 minutes with pumice and B) “Initial” roughness (after water immersion for 7 weeks but before wear) and final roughness after the wear test. The boxes represent the first quartile (Q1) to the third quartile (Q3), the horizontal lines in the box represent the median, the whiskers represent the maximum and minimum values, and “+” represents the mean value (n = 8). Same letters indicate no significant differences (*p*>0.05).

For all materials, mass loss upon abrasion was comparable to volume loss (Fig 6A). Highest mass and volume loss after the brushing test was observed with Gradia (1.05 ± 0.30 wt% and 1.06 ± 0.30 vol%). Mass and volume loss of F1 (0.52 ± 0.05 wt% and 0.49 ± 0.12 vol%) and Z250 (0.47 ± 0.13 wt% and 0.51 ± 0.12 vol%) were comparable. F2 had intermediate values (0.70 ± 0.15 wt% and 0.66 ± 0.16 vol%).

The initial specimen roughness before water immersion was too low to be measurable. After 7 weeks in deionized water, roughness increased significantly in the order of Z250 (0.25 ± 0.05 μm) < Gradia (0.40 ± 0.05 μm) < F1 (1.01 ± 0.25 μm) < F2 (1.66 ± 0.15 μm) (Fig 6B). After toothbrush abrasion, roughness increased in the order Z250 (4.62 ± 0.45 μm) < Gradia (8.56 ± 1.26 μm) < F2 (11.06 ± 1.72 μm) < F1 (14.70 ± 0.62 μm).

**Fig 7:**
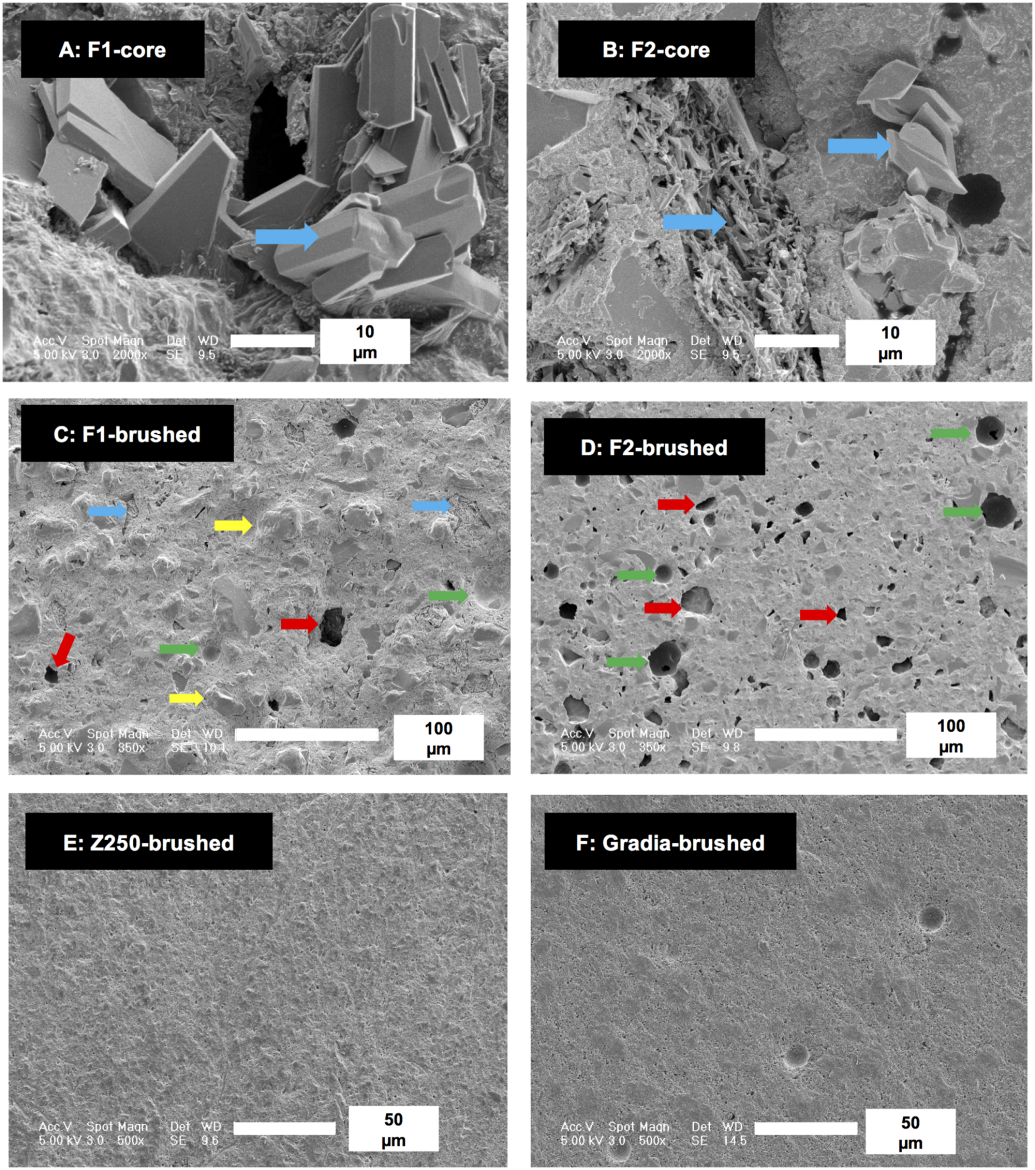
Surface morphology of fracture (core) or worn surfaces. The blue arrows indicate dicalciumphosphate precipitation in fracture surfaces of A) F1 and B) F2 composites. Yellow arrows indicate exposed filler particles whilst green and red arrows indicate holes consistent with loss of PLS and other fillers respectively on brushed surfaces of C) F1 and D) F2. Brushed E) Z250 and F) Gradia surfaces have fewer larger holes.

Upon fracturing wear test samples, needle and plate–like precipitates characteristic of dicalcium phosphates (brushite or monetite) were clearly visible in SEM images of F1 and F2 (Fig 7). From EDX their Ca/P molar ratio was 1 as expected for these phosphates [27]. The phosphates could also be detected in some worn surfaces. F1 and F2 after wear both had areas with larger glass filler particles projecting from the surface and voids consistent in size and shape with loss of larger filler particles (compare Figs 1 and 7). Although F2 in some areas had more holes than F1, it additionally appeared to be more polished. Conversely, SEM images of Z250 and Gradia showed smoother surfaces after wear and fewer obvious large holes.

## Discussion

Dental composites, with added reactive CaP and PLS, using a new monomer system with high molecular weight PPGDMA diluent monomer and a calcium binding monomer (4-META) without amine activator were developed. Monomer conversion, calculated polymerization shrinkage, mass and volume changes and mechanical properties (BFS and BFM) were assessed. Additionally, changes in surface versus bulk composition following SBF immersion or simulated tooth brushing were examined.

### Paste and composite composition and appearance

The level of diluent monomer employed was a similar weight fraction to that of TEGDMA in earlier works [5, 7, 18, 28]. This will, however, equate to a lower molar fraction. Diluent monomer is required in order to enable high filler loading without paste crumbling. 4-META was added at its maximum solubility to promote interaction between the monomer phases, non silane-treated fillers, and dentin. The use of 1 wt% CQ allowed rapid reaction on the top surface. Photobleaching then allows curing of the lower surface in addition to improvement in composite esthetics. Too much CQ would cause too long a delay time before light reaches the lower sample surface. Too little, would slow the reaction rate and drive the need for an amine activator.

Use of a mix of filler sizes enables maximization of filler content as smaller particles fill the voids between close packed larger particles. Use of large particles was included as having all particles below 1 μm substantially lowers maximum powder content which enhances shrinkage. Packable consistency was chosen to prevent “slumping” and facilitate condensation of materials into a prepared tooth cavity.

In the vast majority of studies with remineralizing composites, low solubility calcium phosphates have been employed in an attempt to limit strength reduction caused by high component release [29–33]. In this study, however, high solubility MCPM was included as the remineralizing agent. On the surfaces of the composite, it reacts rapidly with water disproportionating into dicalcium phosphates and phosphoric acid. The acid can etch the tooth. Released calcium ions can then convert the phosphate into low density apatite (Fig 5) which could potentially seal any cracks. In the bulk of the material, the acidic protons will convert TCP into dicalcium phosphates (Fig 7). The greater volume of the dicalcium phosphates compared with that of the reacting CaP has potential to enable expansion [5, 7] and filling of defects or holes in the materials and possibly even that caused by wear.

Sample thickness was fixed at 1 mm as clinicians generally place layers of this depth incrementally to reduce effects of shrinkage and ensure sufficient monomer conversion [34]. Larger diameter specimens were used for gravimetric and wear studies to improve accuracy and provide sufficient surface area for the tooth brush heads. Light exposure for 40 s on both sides was used to ensure high monomer conversion for all specimens.

### Monomer conversion and calculated polymerization shrinkage

High monomer conversion is essential to ensure good mechanical/physical properties and lower the risk of cytotoxic monomer release [29, 35]. Despite amine activator removal, the monomer conversions of the experimental composites (~ 72%) in this study were comparable or higher than that of similar experimental UDMA / TEGDMA based CaP composites (64–72%) [5, 18]. With dimethacrylates, the molecules are likely to first join in long linear chains. Once 50% of the methacrylate groups are polymerized, a slower crosslinking process may occur. Greater crosslinking using PPGDMA instead of TEGDMA as diluent may be a consequence of longer pendant side chains on the linear chains allowing greater mobility for crosslinking. A previous study showed that monomer conversion was decreased upon increasing specimen thickness from 1 to 4 mm due presumably to light scattering [5]. This effect, however, was negligible at 1 mm depth.

A further advantage of using PPGDMA instead of TEGDMA is reduced polymerization shrinkage as the total concentration of double bonds is decreased. The combined high level of glass filler and average monomer molecular weight has enabled production of composites in this study with shrinkage (2.2 vol%) less than that observed with Z250 (2.4 vol%) and Gradia (3.2 vol%) or UDMA/TEGDMA CaP composites (~3 vol%) [5, 18, 36].

### Mass and volume changes

Water uptake can change mass and volume of dental composites [37], thus negatively affect their mechanical and physical properties [38]. This water uptake is, however, required to promote calcium and phosphate release for remineralization or polylysine for antibacterial benefit [39, 40]. Additionally, the resulting volume expansion of the composites was also expected to help compensate the inevitable polymerization shrinkage and relieve the residual shrinkage stress [19, 41].

In agreement with previous studies [5, 7, 18], errors in mass and volume changes are small (<10% of the values). Furthermore, by determining mass and volume change for a prolonged time, it was possible to observe clear differences in later time for different composites despite the small sample number. The mass and volume changes of the experimental composites were comparable or above those observed for packable composites but still lower than those obtained with some flowable commercial materials (~5 vol%) [42]. Water sorption may fill voids within composites increasing mass but not volume [7]. It may also induce expansion of the resin matrix increasing volume more than mass. Water also causes dicalcium phosphates (brushite or monetite) precipitation inside the composites (Fig 7). These low density dicalcium phosphates may force an additional expansion of the polymer network [7, 43].

Mass changes of the composites at late time were comparable with those observed with UDMA/TEGDMA composites with similar levels of CaP, but CHX instead of PLS [5]. The net volume expansion of F1 in the above study was comparable to calculated polymerization shrinkage. This could potentially help to relieve shrinkage stress [19, 44]. This could minimise microgap formation and bacterial microleakage.

### Biaxial flexural strength (BFS) and modulus (BFM)

ISO 4049 flexural strength is determined employing a three-point bending method. Biaxial flexural strengths provide comparable results, but they can additionally be more consistent [45, 46]. ISO 4049 also recommends assessment after 24 hours immersion. In this current study, however, BFS and BFM were obtained after 0, 1 and 4 weeks in solution. These times were chosen as both these properties are known to correlate strongly with water sorption which for CaP-containing composites can be high in the first week of immersion but slows down thereafter [5]. Changes in surrounding solution (water versus SBF) can have considerable effects on surface chemistries of CaP composites [18]. It, however, had no significant effect on the bulk mechanical properties in the current study. This may be due to the surface layer changes with SBF immersion modifying only ~1% of the sample thickness. Additionally, components in the surrounding fluid may have only minor effects on water sorption.

Lower strength of dry specimens of F2 compared to F1 could be a consequence of the active fillers and / or coupling to the monomer phase being weaker than with the silane-treated glasses. Strength and modulus changes with time were greater for F2 between 1 and 4 weeks than with F1 which is consistent with reductions being primarily governed by water sorption. As water sorption is close to equibrium values by 4 weeks further changes after this time are expected to be low. Even with greater CaP and water sorption, the new composites still had higher strength at 4 weeks than that of Gradia (78 MPa) at 1 day [36]. Furthermore, these strengths are higher than that required by ISO 4049 (80 MPa) at 1 day [47]. The current study revealed a strong correlation between reduction in strength and reduction in modulus. This is in agreement with a previous study on similar materials [5]. The reduction in modulus will help to maintain toughness (area under stress–strain curve) upon strength reduction with water sorption.

### Surface apatite formation

Level, type, and rate of apatite precipitation on the surface of materials depends on the pH and ion concentration of the environment [48]. A previous study showed level of apatite precipitation was proportional to time and CaP level [28]. Furthermore, precipitatation also increased upon PLS addition [18]. These findings are consistent with the results in above studies. The Ca to P molar ratio of the apatite layer of F2, at 4 weeks, indicated that the precipitates were calcium-deficient hydroxyapatite which is commonly found in biological apatite [49]. The thicker layers of F2 at 4 weeks (~15 μm) are of comparable size to the gaps that could form due to polymerization shrinkage (~10 μm) [50]. Furthermore, a study indicated that restorative materials that promote surface apatite formation could remineralize the demineralized dentin [8].

### Accelerated wear test

This study employed a simple and low cost apparatus for dental wear assessment. In pilot studies, tooth paste was used as an abrasive medium but no significant changes were detected. Hence, large particle pumice was chosen to accelerate the abrasive wear. Brushing with pumice for 20 min was sufficient to allow significant quantitative and qualitative comparisons between materials. This protocol, however, complicates direct comparison with other published studies.

### Mass and volume loss

Wear of a dental composite is governed by several factors such as the testing methods, filler sizes and compositions, filler-polymer matrix interaction, and the degree of monomer conversion [51]. The water absorbed into a composite may also soften the polymer network and weaken the interfacial bond strength between matrix and fillers [52]. Tooth brushing generally wears first the softer polymer matrix and then loosens the filler particles [53].

Similarity in mass versus volume loss of the composites suggests levels of abraded polymer and higher density filler are comparable. This is to be expected if depth of wear is much greater than filler size. From the mass and volume loss (~1%), estimated wear depth is ~ 10 μm. This is much larger than the average filler diameter of Gradia (0.85 μm) and Z250 (0.65 μm) [54, 55]. In the experimental materials, greater loss of MCPM could compensate lower loss of glass filler. This could ensure comparable loss of lower density polymer versus higher density filler.

The highest mass and volume loss observed from Gradia could be due to the incorporation of pre-polymerized fillers [56]. These fillers may be easily abraded from the resin matrix due to the lack of surface reactive groups to bind with the polymerizing resin matrix [36, 57]. The greater mass and volume loss in F2 compared to F1 could be due to higher water sorption at greater CaP filler level. Despite the high water sorption, the experimental composites exhibited mass and volume loss lower than Gradia and not far off from Z250. This could be due to their higher monomer conversions.

### Surface roughness

The increase of R_a_ after water immersion could be due to some dissolution of reactive CaP fillers and/or low level reprecipitation as apatites or brushite. Upon abrasion, R_a_ can increase due to inorganic fillers standing out and poorly bound active filler particles detaching. Commercial composites exhibited lower R_a_ than experimental composites presumably because of their smaller average filler diameter. With experimental materials, brushing could also remove dicalcium phosphate and water soluble PLS that were poorly bound to the resin matrix. This could generate holes with diameters comparable with those of the particles.

The tooth brushing wear testing may expose holes underneath the surface generated by PLS and MCPM that would have partially dissolved upon ageing in water for 7 weeks. These holes would be expected to be more evident in F2 than F1. R_a_ of F2 was, however, lower than that of F1. This could be due to dicalcium phosphates dissolving and reprecipitating in small cracks or irregularities, thereby helping to smooth the surface of reactive CaP composites.

Experimental composites exhibited high surface roughness upon accelerated tooth brushing wear which could potentially enhance bacterial accumulation. It is, however, expected that antibacterial polylysine, which can be readily released from the composite surface, could help to reduce the formation of biofilm. Additionally, over longer times than in the accelerated wear test, they might be more effectively smoothed by surface apatite precipitation.

## Conclusion

Despite amine activator removal, the PPGDMA containing composites had high monomer conversion in comparison with other dental composites. The increase of CaP and PLS led to higher water sorption, reduced strength, and increased mass and volume loss upon wear. These experimental material properties, however, remained comparable with commercial formulations. In addition, active fillers enhanced hygroscopic expansion and surface apatite formation which are expected to help compensate polymerization shrinkage, seal gaps due to the shrinkage, and remineralize the demineralized dentin. The increase of reactive fillers also enabled a reduction in surface roughness upon wear.

## Supporting information

S1 File Raw data. Experimental and commercial dental composites raw data.
(XLSX)
